# An Upsurge of Measles Cases in Mali—a Consequence of Pandemic-associated Disruption in Routine Immunization

**DOI:** 10.1093/ofid/ofae154

**Published:** 2024-03-19

**Authors:** Nginache Nampota-Nkomba, Adama Mamby Keita, Jane Juma, Diakaridia Sidibe, Nana Kourouma, Seydou Sissoko, Fadima Cheick Haidara, Cheick Tidiane Traore, Cheick Bougadari Traore, Awa Traore, Brigitte Gaume, Samba Ousmane Sow, Karen L Kotloff, Milagritos D Tapia

**Affiliations:** Center for Vaccine Development and Global Health, University of Maryland School of Medicine, Baltimore, Maryland, USA; Centre pour le Développement des Vaccins-Mali, Bamako, Mali; Centre pour le Développement des Vaccins-Mali, Bamako, Mali; Centre pour le Développement des Vaccins-Mali, Bamako, Mali; Centre pour le Développement des Vaccins-Mali, Bamako, Mali; Centre pour le Développement des Vaccins-Mali, Bamako, Mali; Centre pour le Développement des Vaccins-Mali, Bamako, Mali; Direction Générale de la Santé et de l’Hygiène Publique, Ministry of Health and Social Development, Bamako, Mali; Histopathology Laboratory, Centre Hospitalo-Universitaire du Point G, Bamako, Mali; Centre pour le Développement des Vaccins-Mali, Bamako, Mali; Center for Vaccine Development and Global Health, University of Maryland School of Medicine, Baltimore, Maryland, USA; Centre pour le Développement des Vaccins-Mali, Bamako, Mali; Center for Vaccine Development and Global Health, University of Maryland School of Medicine, Baltimore, Maryland, USA; Center for Vaccine Development and Global Health, University of Maryland School of Medicine, Baltimore, Maryland, USA

**Keywords:** child health, measles, measles mortality, measles vaccines, vaccination coverage

## Abstract

Measles deaths highlight immunization program gaps. In the Child Health and Mortality Prevention Surveillance study in Mali, we observed a rise in under-5 measles-related deaths in 2022 that corresponded with increased measles cases at the same time and a decline in measles vaccine coverage in Mali in 2020.

As a highly contagious, vaccine-preventable disease with an estimated reproductive number of 12–18, the occurrence of measles and measles-related fatalities within a community serves as a sensitive indicator of suboptimal vaccination coverage [1, 2]. In 2019, approximately 69 800 children under the age of 5 years died from measles, with a disproportionate burden falling on sub-Saharan Africa (SSA), accounting for 78% of the global deaths [3].

Despite collective commitments from all World Health Organization (WHO) regions to eliminate measles by administering 2 doses of measles-containing vaccine (MCV) at age 9 and 15 months in areas with active measles transmission, and at 12 and ≥15 months in low-risk settings [4, 5] no region has experienced sustained elimination. Nevertheless, 83 individual countries achieved the goal in 2022 [6]. Achieving 95% vaccination coverage for both doses is imperative to interrupt measles transmission and attain herd immunity [7, 8].

The occurrence of measles deaths indicates that a substantial number of cases are occurring within the community [9]. However, incidence rates are difficult to estimate because of variable case fatality rates resulting from factors such as MCV dose receipt, nutritional status, age, and wealth index. Here, we describe measles-related deaths that occurred in the Child Health and Mortality Prevention Surveillance (CHAMPS) study in Bamako, Mali, in the context of local measles disease epidemiology, the pandemic of COVID-19, and MCV coverage in Mali during the same period.

## METHODS

The CHAMPS network is a surveillance system to track the causes of death for stillbirths and children younger than 5 years of age at demographically characterized sites in Mali, Ethiopia, Sierra Leone, Mozambique, Kenya, South Africa, and Bangladesh, and the study methods were previously described [10–13]. Briefly, within a subset of deaths in which parental consent is obtained, we conduct minimally invasive tissue sampling to obtain biopsies of internal organs and collect whole blood, cerebrospinal fluid, as well as nasal, oral, pharyngeal, and rectal swabs. The specimens undergo evaluation through quantitative polymerase chain reaction using multiplexed TaqMan Array Cards, culture, histopathology with special stains, and immunohistochemistry as appropriate. Clinical history is gathered from medical records and verbal autopsies [14]. These data are reviewed by a panel of expert clinicians, pathologists, epidemiologists, and laboratorians using a standardized Determination of Cause of Death adjudication process in which the causal chain is determined per International Classification of Diseases and the WHO death certificate criteria, including the designation of an underlying and intermediate cause of death [15, 16]. In Mali, CHAMPS cause-of-death assessments are conducted in Bamako, covering Djikoroni-para and Banconi from 2017, with Sebenikoro added in 2022.

To provide a contextual backdrop for the CHAMPS measles deaths in Mali, we conducted a descriptive study by reviewing routine data from the Mali Ministry of Health, focusing on laboratory-confirmed measles cases detected during routine surveillance and MCV coverage. Additionally, we examined standardized measles incidence and MCV coverage rates from the WHO and United Nations International Children's Emergency Fund (UNICEF) [17]. In Mali, the administration of the first dose of MCV (MCV1) occurs at 9 months, followed by a second dose (MCV2) between 15 and 18 months of age.

## RESULTS

Between August 2017 and December 2022, CHAMPS-Mali conducted minimally invasive tissue sampling on 562 children. Among these, 485 underwent the Determination of Cause of Death process, whereas 77 are pending adjudication. Eight (1.6%) children were identified as having measles in the causal chain of their death distributed across the years 2017 (1 case), 2018 (1 case), 2021 (2 cases), and 2022 (4 cases). The children's ages ranged from 4 months to 5 years; 5 cases were female. Of the 6 children who were age-eligible for MCV, 3 were unvaccinated, and vaccination status for the remaining 3 was unknown. Two children contracted measles at 4 and 8 months of age, before the age of routine measles vaccination. For all the children, the immediate cause of death was sepsis or pneumonia with measles infection as an underlying or morbid condition. All children had malnutrition as a morbid, underlying, or other significant condition and 5 children had concurrent gastroenteritis. The cases are summarized in Table 1.

**Table 1.** Measles Deaths in CHAMPS—Clinical Case Summaries

Date of Death	Age	Sex	HIV DNA PCR	Nutritional Status^a^	MCV Status	Clinical Presentation	Measles Positive Tests^b^	Pathology Report	Immediate Cause Of Death^c^	Morbid Condition^c^	Underlying Conditions/Other Significant Conditions^c^
November 2017	2 y, 11mo	F	Neg	WHZ: −4.86 WAZ: −5.28 LAZ: −3.53 HCAZ: −2.45 MUAC Z: −4.18	Unk	Not available	Blood CSF Lung ONP	Lung: neutrophilic interstitial pneumonitis. Evidence of *Escherichia*. coli and *Klebsiella pneumoniae* in vessels Liver: moderate steatosis (small and large droplet) CNS: intravascular fibrin thrombi and evidence of *E coli* and *K pneumoniae*	Sepsis (*E coli*)	Sepsis (*K pneumoniae*)	Measle/severe malnutrition
June 2018	3 y, 9mo	F	Neg	WHZ: −2.41 WAZ: −0.85 LAZ: 1.25 HCZ: −0.12 MUAC Z: −0.08	No	Fever, cough, difficulty breathing, rash, diarrhea, vomiting, oral mycosis, night sweats, loss of consciousness, convulsion, agitation, nightmares, chest pain, dysphagia, odynophagia	Blood CSF Lung ONP	Lung: bronchopneumonia and aspiration pneumonia with mixed bacteria and gastrointestinal contents. Evidence of measles virus, *Streptococcus* species and *Hemophilus* influenzae. Liver: mild sinusoidal leukocytosis	Polymicrobial pneumonia (*H influenzae, S pneumoniae*)	…	Measles/ Rickets, Enteritis, use of traditional medication
September 2021	1 y, 4mo	M	Neg	WHZ: −3.88 WAZ: −3.22 LAZ: 0.95 HCZ: −2.9 MUAC Z: −3.22	Unk	Fever, cough, difficulty breathing, rash, diarrhea, vomiting, oral mycosis, night sweats, loss of consciousness, convulsion, discoloration of eyes, distended abdomen.	Blood Lung ONP	Lung: interstitial pneumonitis, and bronchopneumonia. Evidence of measles, *Klebsiella* species and *Streptococcu*s species. Liver: mild congestion and large droplet steatosis, focal intravascular bacteria.	Polymicrobial sepsis (*K pneumoniae, S. aureus, Moraxella catarrhalis*)	Bacterial and viral pneumonia (*K pneumoniae, S aureus, M catarrhalis*, measles). Gastroenteritis	Measles/severe malnutrition
November 2021	∼2y	F	Neg	WHZ: −2.64 WAZ: NA HAZ: NA HCZ: NA MUAC Z: NA	No	Fever, cough, pruritis, boils on the skin, diarrhea, edema of the limbs, face swelling, anal prolapse, foot ulcer, weight loss, polydipsia	Blood ONP	Lung: inadequate sample Liver: severe steatosis and sinusoidal leukocytes	Polymicrobial sepsis (*E coli/Shigella,* *Pseudomonas aeruginosa, S pneumoniae,* *M catarrhalis*)	Measles Shigellosis with rectal prolapse Diarrhea	Kwashiorkor/ bullous impetigo
February 2022	<5y	F	Neg	WHZ: −2.9 WAZ: NA HAZ: NA HCZ: NA MUAC Z: NA	Unk	Not available	Blood CSF Lung ONP	Lung: interstitial pneumonitis, and bronchopneumonia. Evidence of *Klebsiella* species and *Streptococcus* species. Liver: mild congestion and mild steatosis	Pneumonia (*K pneumoniae and S pneumoniae*)	Moderate malnutrition	Measles/disseminated herpes simplex virus 1 infection
March 2022	11mo	M	Unk	WHZ: −3.22 WAZ: −4.12 HAZ: −3.64 HCZ: −3.53 MUAC Z: −1.53	No	Fever, cough, difficulty breathing, rash, diarrhea, night sweats, runny nose, sunken eyes.	Blood Lung ONP	Lung: interstitial pneumonitis, and bronchopneumonia. Evidence of *Streptococcus* species. Liver: mild congestion and severe steatosis. CNS: intravascular leukocytosis	Pneumonia (*S pneumoniae and Group A streptococcus* [GAS])	Sepsis *(Staphylococcus aureus, S pneumoniae*, GAS, and *E coli/Shigella*) Severe malnutrition Diarrhea	Measles
May 2022	4mo	M	Unk	WHZ: −3.73 WAZ: −5.03 HAZ: −3.68 HCZ: −2.07 MUAC: −5.58	NA	Fever, cough	Blood Lung	Lung: bronchopneumonia. Evidence of cytomegalovirus (CMV), gram-variable rods and *Pneumocystis jiroveci* (PJP). Liver: mild congestion, severe steatosis, and sinusoidal leukocytosis CNS: intravascular leukocytosis	Polymicrobial sepsis (*S pneumoniae, P aeruginosa, K* *pneumonia, M catarrhalis, Acinetobacter* *baumanii, E coli/Shigella*)	Disseminated measles. Enteric infection (Norovirus, campylobacter) Pneumonia (*S pneumoniae measles*, PJP)	Severe malnutrition (acute on chronic) Immunodeficiency of unknown cause
May 2022	8mo	F	Unk	WHZ: −3.51 WAZ: −2.24 LAZ: 0.69 HCZ: −0.68 MUAC Z: −2.95	NA	Cough, difficulty breathing, rash, diarrhea	Blood Lung ONP	Lung: interstitial pneumonitis. Evidence of *Hemophilus* species Liver: mild congestion and mild steatosis.	Pneumonia *(H influenzae*)	Moderate malnutrition Diarrhea	Measles

Abbreviations: CSF, cerebrospinal fluid; HCZ, head circumference *Z* score; LAZ, length for age *Z* score; MUACZ, mean upper arm circumference *Z* score; NA, nonapplicable; Neg, negative; ONP, oral, nasal, and pharyngeal specimen; Unk, unknown; WAZ, weight for age *Z* score; WHZ, weight for height *Z* score.

^a^Nutritional status *Z* score interpretation: Mild malnutrition = −1.0 to −1.9; moderate malnutrition = −2.0 to −2.9; and severe malnutrition = ≥−3.0.

^b^Tested through quantitative polymerase chain reaction using multiplexed TaqMan Array Cards, culture, histopathology with special stains, or immunohistochemistry.

^c^Causal death pathways as determined through the Determination of Cause of Death process: immediate cause of death = disease or condition that directly preceded or directly led to death; morbid condition = disease or condition that was the intermediate cause of death; underlying condition = disease or injury that initiated the train of events leading directly to death; other significant disease = condition not directly associated or in the causal chain of death, but still contributed to the death.

**Figure 1.**
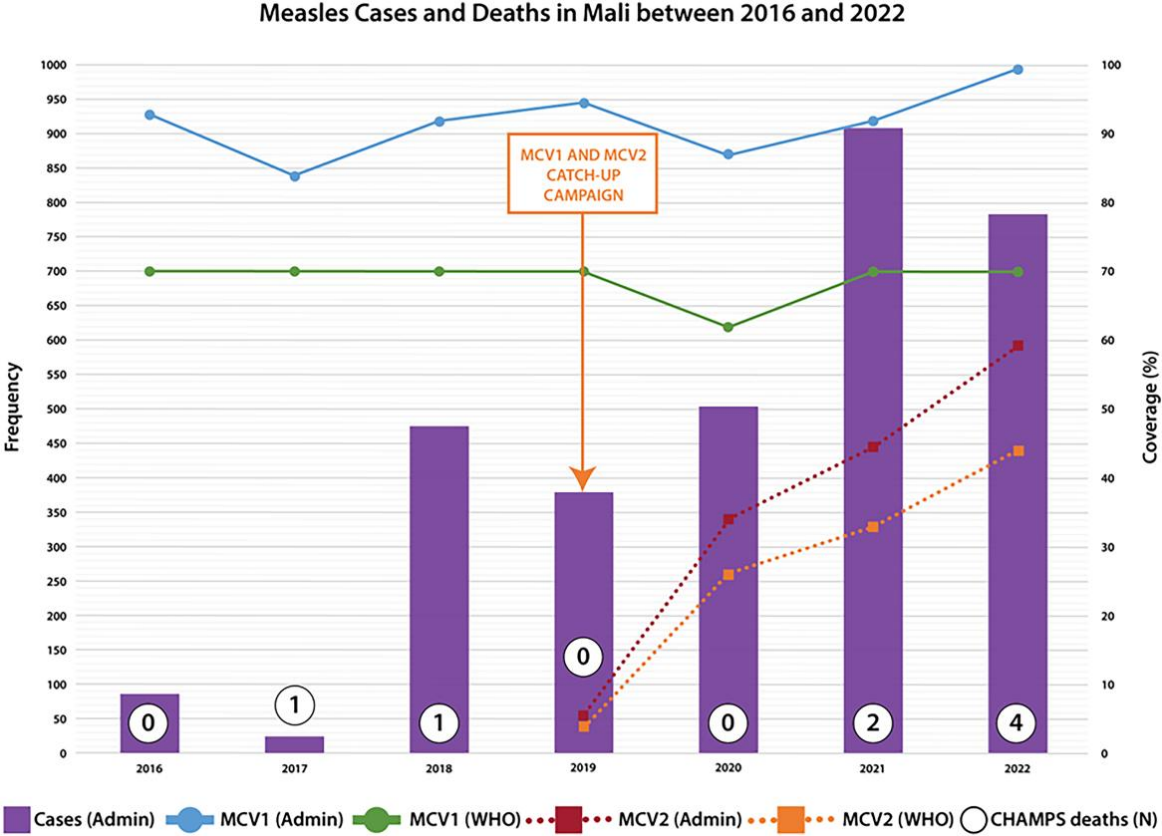
Reported measles cases and measles-containing vaccine coverage from Mali Ministry of Health administrative data. MCV2 was first introduced in 2019 in Mali. MCV1, measles containing vaccine 1; MCV2, measles containing vaccine 2; WHO, World Health Organization/United Nations International Children's Emergency Fund estimates of Mali measles-containing vaccine coverage.

National data across all age groups in Mali for the same period showed a similar trend of increased frequency of laboratory-confirmed measles after 2020 (Figure 1). The reported measles cases decreased from 87 in 2016 to 26 in 2017 but then increased nearly 20-fold to 476 in 2018. The numbers remained stable thereafter, before doubling to 908 in 2021 and settling at 748 in 2022. The WHO/UNICEF estimates for measles incidence per 1 million population reflected this surge, rising from 1.3 cases in 2017 to 22.1 in 2019 and reaching 94.7 in 2021 before dropping to 33.9 in 2022 [17].

Correspondingly, the periods of increased measles cases in 2017–2018 and 2020–2021 aligned with declines in MCV1 coverage (Figure 1). Mali's Ministry of Health reported consistent national MCV1 coverage exceeding 90% since 2016, except for dips in 2017 (83%) and 2020 (87%). WHO/UNICEF standardized estimates of MCV1 coverage in Mali mirrored this, holding steady at 70% since 2016 but dropping to 62% in 2020 [17]. Since the introduction of MCV2 into routine immunization in 2019, coverage has risen from 5.5% to 59% in 2022, as indicated by local data, aligning closely with WHO/UNICEF estimates of 4% and 44%, respectively, for the same period (Figure 1) [17]. Mali had 2 catch-up measles vaccination campaigns around our study period in 2015 and 2019 and had administrative coverage rates of 112.5% and 108.9%. Follow-up national administrative surveys reported coverage rates of 93.5% and 82.7%, respectively.

## DISCUSSION

We report a notable increase in measles-related deaths among children younger than age 5 years old in 2022 compared with previous years in our study, corresponding to a surge in measles cases and a decline in MCV1 vaccine coverage in Mali. The deceased children were mainly very young with 25% being too young to be vaccinated, unvaccinated, and malnourished, and exhibiting measles-compatible symptoms complicated by bacterial pneumonia, gastroenteritis, and sepsis [18, 19]. Measles mortality rates are often challenging to accurately estimate because of the lack of mortality data, and varying reporting practices depending on factors such as community versus hospital deaths, age, nutritional status, associated complications, and country. Moreover, they are often underestimated in low- and middle-income settings [20]. Nonetheless, our 4 observed deaths in 2022 corroborate a manifold rise in measles community circulation in Bamako from 2019 to 2022 .

The increase in measles cases in 2022 was preceded by a 6% and 3% drop in WHO/UNICEF and Mali estimates of MCV coverage in 2020, pointing to immunization gaps as a potential contributing factor to the outbreak. The 2 notable events in 2020 were the COVID-19 pandemic, which was first detected in Mali in March of that year, and political instability that heightened during the national elections of 2020 [21, 22]. These 2 events likely led to insecurity and decreased vaccination session attendance, potentially increasing the pool of susceptible children to measles [23]. This is supported by global data that shows declination in routine childhood immunization coverage and outreach services globally during 2020 [24]. Notably, the MCV1 coverage rate reported by the WHO/UNICEF was lower than that reported by the Mali Ministry of Health. WHO/UNICEF estimates were based on coverage surveys and not country estimates because of inconsistencies in the country's data [23]. Still, the trend of the coverage is similar.

The resurgence of measles after the COVID-19 pandemic was not unique to Mali, with similar trends observed in other SSA countries. Liberia saw a >6-fold increase in its incidence of measles cases per 1 million population from 214 to 1565.2, which was associated with a drop in MCV1 coverage from 68% in 2019 to 61% in 2021 [17]. Other SSA countries that saw a significant increase in their measles incidence were Somalia, South Africa, Ethiopia, and Côte d'Ivoire, and the outbreaks were associated with MCV coverage rates that ranged from 46% to 85% [17, 25, 26]. Additionally, measles catch-up vaccination campaigns were delayed because of the COVID-19 pandemic [27]. In fact, in the general SSA region between 2019 and 2021, estimated annual measles cases and deaths increased by 22% and 8%, respectively, MCV1 coverage decreased from 70% to 68%, the number of countries with ≥95% coverage decreased from 6 to 3, and no country in the WHO African region had received verification of measles elimination [28]. This set back the measles elimination targets for the continent by several years [29]. Similar trends have been observed globally, and none of the WHO regions has recovered MCV coverage levels from 2019 [30].

Our data on measles deaths highlight the risk of measles faced by young infants during the “window of vulnerability” when maternal antibodies wane before vaccine eligibility at 9 months. It was previously shown in Mali that many infants are susceptible to measles before 9 months of age [31, 32]. However, routine immunization is not recommended for these younger children because of lower immunogenicity; therefore, achieving herd immunity is paramount to protect these vulnerable children from infection. Unfortunately, except for 2022, Mali's MCV1 coverage has fallen short of the 95% elimination vaccination coverage rate, with MCV2 coverage lagging further behind. Despite measles catch-up campaigns in 2015 and 2019, our data suggest ongoing community transmission. Further catch-up campaigns are needed, especially after 2020 when the rate of accumulation of susceptible cases may have been higher than previous years [24, 33]. Regular catch-up campaigns, including during immunization program disruptions, have proven effective in filling immunization gaps and controlling measles [34–37]. Our study captures measles deaths that may have otherwise gone unnoticed during routine surveillance. The main limitation is that it is descriptive and cannot establish a statistical association between the drop in MCV coverage and rise in measles cases. We do not have data on the reasons for missed vaccination in the CHAMPS measles cases. Nonetheless, our results tally with local data on measles transmission. The trends we report align with WHO/UNICEF standardized data and are consistent with measles outbreaks occurring in other similar settings.

## CONCLUSION

Measles continues to circulate among infants and young children in Mali, as evidenced by our mortality surveillance, particularly following the decline in MCV coverage in 2020. It is imperative to implement catch-up immunization efforts to reach children who were inadvertently missed during routine vaccination activities.

## References

[1] John J . Measles: a canary in the coal mines? Indian J Pediatr 2016; 83:195–6. 26809769 10.1007/s12098-015-2004-z

[2] Guerra FM , Bolotin S, Lim G, et al The basic reproduction number (R0) of measles: a systematic review. Lancet Infect Dis 2017; 17:e420–8. 28757186 10.1016/S1473-3099(17)30307-9

[3] Global Burden of Disease Collaborative Network. Global burden of disease, measles—level 3 cause. 2020. Available at: https://www.healthdata.org/results/gbd_summaries/2019/measles-level-3-cause

[4] World Health Organization . Measles vaccines: WHO position paper—April 2017. Wkly Epidemiol Rep 2017; 17:205–28. 10.1016/j.vaccine.2017.07.066 28760612

[5] World Health Organization . Immunization agenda 2030. 2020. Available at: https://www.who.int/docs/default-source/immunization/strategy/ia2030/ia2030-document-en.pdf

[6] Minta AA , Ferrari M, Antoni S, et al Progress toward measles elimination—worldwide, 2000–2022. MMWR Morb Mortal Wkly Rep 2023; 72:1262–8. 37971951 10.15585/mmwr.mm7246a3 PMC10684353

[7] World Health Organization . Measles and rubella strategic framework. 2021 Available at: https://www.who.int/publications/i/item/measles-and-rubella-strategic-framework-2021-2030

[8] Moss WJ , Strebel P. Biological feasibility of measles eradication. J Infect Dis 2011; 2041(Suppl 1):S47–53. 10.1093/infdis/jir065 PMC3112320 21666201

[9] Sbarra AN , Mosser JF, Jit M, et al Estimating national-level measles case–fatality ratios in low-income and middle-income countries: an updated systematic review and modelling study. Lancet Glob Health 2023; 11:e516–24. 36925172 10.1016/S2214-109X(23)00043-8 PMC10030458

[10] Rakislova N , Fernandes F, Lovane L, et al Standardization of minimally invasive tissue sampling specimen collection and pathology training for the Child Health and Mortality Prevention Surveillance Network. Clin Infect Dis 2019; 69:S302–10. 31598667 10.1093/cid/ciz565 PMC6785668

[11] Martines RB , Ritter JM, Gary J, et al Pathology and telepathology methods in the Child Health and Mortality Prevention Surveillance Network. Clin Infect Dis 2019; 69:S322–32. 31598668 10.1093/cid/ciz579

[12] Diaz MH , Waller JL, Theodore MJ, et al Development and implementation of Multiplex TaqMan array cards for specimen testing at Child Health and Mortality Prevention surveillance site laboratories. Clin Infect Dis 2019; 69:S311–21. 31598666 10.1093/cid/ciz571 PMC7108207

[13] Salzberg NT , Sivalogan K, Bassat Q, et al Mortality surveillance methods to identify and characterize deaths in Child Health and Mortality Prevention surveillance network sites. Clin Infect Dis 2019; 69:S262–73. 31598664 10.1093/cid/ciz599 PMC6785672

[14] World Health Organization . WHO. 2016. Verbal autopsy standards. Available at: https://www.who.int/standards/classifications/other-classifications/verbal-autopsy-standards-ascertaining-and-attributing-causes-of-death-tool

[15] Blau DM , Caneer JP, Philipsborn RP, et al Overview and development of the child health and mortality prevention surveillance determination of cause of death (DeCoDe) process and DeCoDe diagnosis standards. Clin Infect Dis 2019; 69(Suppl 4):S333–41. 31598661 10.1093/cid/ciz572 PMC6785670

[16] World Health Organization . The WHO application of ICD-10 to deaths during the perinatal period: ICD-PM. WHO. 2016 Available at: https://www.who.int/publications/i/item/9789241549752

[17] World Health Organization . WHO Immunization Data Portal. 2022. WHO immunization portal. Available at: https://immunizationdata.who.int/pages/coverage/mcv.html

[18] Alves Graber EM , Andrade FJ, Bost W, Gibbs MA. An update and review of measles for emergency physicians. J Emerg Med 2020; 58:610–5. 32241708 10.1016/j.jemermed.2020.02.007

[19] Perry RT , Halsey NA. The clinical significance of measles: a review. J Infect Dis. 2004; 189(Suppl 1):S4–16. 15106083 10.1086/377712

[20] Cairns KL , Nandy R, Grais RF. Challenges in measuring measles case fatality ratios in settings without vital registration. Emerg Themes Epidemiol 2010; 7:4. 20642812 10.1186/1742-7622-7-4 PMC2918600

[21] Human Rights Watch. World Report 2021—Mali. 2021. Available at: https://www.hrw.org/world-report/2021/country-chapters/mali

[22] Ahmed A , Ly MA, Diarra BA, et al Challenges to the implementation and adoption of physical distancing measures against COVID-19 by internally displaced people in Mali: a qualitative study. Confl Health 2021; 15:88. 34863236 10.1186/s13031-021-00425-x PMC8642860

[23] World Health Organization . Mali: WHO and UNICEF estimates of immunization coverage: 2022 revision. 2022. Available at: https://data.unicef.org/wp-content/uploads/cp/immunisation/mli.pdf.

[24] Shet A , Carr K, Danovaro-Holliday MC, et al Impact of the SARS-CoV-2 pandemic on routine immunisation services: evidence of disruption and recovery from 170 countries and territories. Lancet Glob Health 2022; 10:e186–94. 34951973 10.1016/S2214-109X(21)00512-X PMC8691849

[25] Oduoye MO , Zuhair V, Marbell A, et al The recent measles outbreak in South African region is due to low vaccination coverage. What should we do to mitigate it? New Microbes New Infect 2023; 54:101164. 37455850 10.1016/j.nmni.2023.101164 PMC10344677

[26] Nchasi G , Paul IK, Sospeter SB, Mallya MR, Ruaichi J, Malunga J. Measles outbreak in sub-Saharan Africa amidst COVID-19: a rising concern, efforts, challenges, and future recommendations. Ann Med Surg (Lond) 2022; 81:104264. 35937634 10.1016/j.amsu.2022.104264 PMC9339085

[27] Ho LL , Gurung S, Mirza I, et al Impact of the SARS-CoV-2 pandemic on vaccine-preventable disease campaigns. Int J Infect Dis 2022; 119:201–9. 35398300 10.1016/j.ijid.2022.04.005 PMC8985404

[28] Masresha BG , Hatcher C, Lebo E, et al Progress toward measles elimination - African region, 2017-2021. MMWR Morb Mortal Wkly Rep.;72:985–991. 10.15585/mmwr.mm7236a3 PMC10495184 37676836

[29] World Health Organization Africa . Measles elimination by 2020: a strategy for the African Region. 2011. Available at: https://www.afro.who.int/sites/default/files/sessions/working_documents/AFR-RC61-8-Measles-elimination-by-2020—strategy-forthe-African-Region—with-Resolution.pdf

[30] Minta AA , Ferrari M, Antoni S, et al Progress toward regional measles elimination—worldwide, 2000–2021. MMWR Morb Mortal Wkly Rep 2022; 71:1489–95. 36417303 10.15585/mmwr.mm7147a1 PMC9707362

[31] Tapia MD , Sow SO, Medina-Moreno S, et al A serosurvey to identify the window of vulnerability to wild-type measles among infants in rural Mali. Am J Trop Med Hyg 2005; 73:26–31. 16014826

[32] Dixon MG , Tapia MD, Wannemuehler K, et al Measles susceptibility in maternal-infant dyads—Bamako, Mali. Vaccine 2022; 40:1316–22. 35101263 10.1016/j.vaccine.2022.01.012 PMC8861573

[33] Kreidl P , Ammerer D, Würzner R, Luckner Hornischer A, Von Laer D, Borena W. Measles elimination: identifying susceptible sub-populations to tailor immunization strategies. Viruses 2019; 11:765. 31434243 10.3390/v11080765 PMC6723762

[34] Clarke A , Blidi N, Yokie J, et al Strengthening immunization service delivery post Ebola virus disease (EVD) outbreak in Liberia 2015–2017. Pan Afr Med J 2019; 33:5. 10.11604/pamj.supp.2019.33.2.17116 PMC6675927 31402965

[35] Marin M , Nguyen HQ, Keen J, et al Importance of catch-up vaccination: experience from a varicella outbreak, Maine, 2002–2003. Pediatrics 2005; 115:900–5. 15805362 10.1542/peds.2004-1162

[36] Simone B , Balasegaram S, Gobin M, et al Evaluation of the measles, mumps and rubella vaccination catch-up campaign in England in 2013. Vaccine 2014; 32:4681–8. 24996125 10.1016/j.vaccine.2014.05.077

[37] Zhang RQ , Li HB, Li FY, Han LX, Xiong YM. Epidemiological characteristics of measles from 2000 to 2014: results of a measles catch-up vaccination campaign in Xianyang, China. J Infect Public Health 2017; 10:624–9. 28254459 10.1016/j.jiph.2017.02.005

